# Causal, Bayesian, & non-parametric modeling of the SARS-CoV-2 viral load distribution vs. patient’s age

**DOI:** 10.1371/journal.pone.0275011

**Published:** 2022-10-05

**Authors:** Matteo Guardiani, Philipp Frank, Andrija Kostić, Gordian Edenhofer, Jakob Roth, Berit Uhlmann, Torsten Enßlin

**Affiliations:** 1 Max Planck Institute for Astrophysics, Garching, Germany; 2 Fakultät für Physik, Ludwig-Maximilians-Universität München, Munich, Germany; 3 Excellence Cluster ORIGINS, Garching, Germany; 4 Süddeutsche Zeitung, Munich, Germany; Universitatsklinikum Schleswig Holstein Campus Lubeck, GERMANY

## Abstract

The viral load of patients infected with SARS-CoV-2 varies on logarithmic scales and possibly with age. Controversial claims have been made in the literature regarding whether the viral load distribution actually depends on the age of the patients. Such a dependence would have implications for the COVID-19 spreading mechanism, the age-dependent immune system reaction, and thus for policymaking. We hereby develop a method to analyze viral-load distribution data as a function of the patients’ age within a flexible, non-parametric, hierarchical, Bayesian, and causal model. The causal nature of the developed reconstruction additionally allows to test for bias in the data. This could be due to, e.g., bias in patient-testing and data collection or systematic errors in the measurement of the viral load. We perform these tests by calculating the Bayesian evidence for each implied possible causal direction. The possibility of testing for bias in data collection and identifying causal directions can be very useful in other contexts as well. For this reason we make our model freely available. When applied to publicly available age and SARS-CoV-2 viral load data, we find a statistically significant increase in the viral load with age, but only for one of the two analyzed datasets. If we consider this dataset, and based on the current understanding of viral load’s impact on patients’ infectivity, we expect a non-negligible difference in the infectivity of different age groups. This difference is nonetheless too small to justify considering any age group as noninfectious.

## Introduction

Children do not seem to be major drivers in the transmission of Severe Acute Respiratory Syndrome Coronavirus 2 (SARS-CoV-2) in the general population [1]. However, the exact degree to which children and adolescents get infected by, and are able to transmit the virus is not yet well known. Their role in the community spread depends on their susceptibility, symptoms, viral load, social contact patterns, behavior, and existing mitigation strategies as schools and daycares closings. Among all these variables, the viral load plays a fundamental role. The viral load might help to predict disease severity [2] and mortality [3–5] and can serve as a proxy for the infectivity of the patient [6–8]. The severity of a disease, its infectivity, and its mortality are certainly fundamental parameters that must be considered when deciding on best-practice preventative measures to fight the pandemic spread. Research in this direction can enable truly data-driven policymaking like, for example, school openings and focused lockdowns. For this scope, it is important to understand how the viral load depends on the patients’ age.

In this work, we examine viral load as a proxy for infectivity. We reanalyze the age-stratified viral load data from Jones et al. [9] in order to better understand the actual relation between these variables. We do this in the hope of gaining insight into fundamental differences reported in the literature regarding the relationship between viral load and age. We achieve this goal by developing a flexible, non-parametric, causal, and Bayesian model to reconstruct the conditional probability density function (PDF) of the viral load given the patient’s age. The developed method is a second central result of this work: it can be applied to future studies on SARS-CoV-2, to similar data from other diseases, and also to many causally connected quantities in very different contexts.

The non-parametric PDF reconstruction is regularized by mild assumptions on the smoothness of the underlying statistical processes. In particular, we assume that the log-densities are Gaussian-process realizations, drawn with an a priori flexible correlation kernel parametrized by a Matérn family correlation function. The parameters of this correlation function are then inferred along with the PDF through a variational inference algorithm. To achieve this, we adapt methods developed for information field theory, the information theory for fields [10, 11]. In this context, fields are understood as spatially varying (physical) quantities. The reconstructed PDFs are regarded as scalar fields whose values are defined at each point of the two-dimensional space spanned by age and viral load and represent the probability of observing a given combination of age and viral load. The toolkit of information field theory has proven itself to be successful in a wide range of applications, ranging from 3D tomography [12], over time-resolved astronomical imaging [13], to causality inference [14].

### Outline

The rest of this work is structured as follows: in Sec. Related work, we discuss the state of the art of the research on the assessment of the viral load-vs-age dependence. In Sec. Model design, we motivate the need for a causal description and show how this description is built into the model and the inference scheme we adopt. In Sec. Data, we describe how the data has been acquired and processed. We then outline the main results of our study in Sec. Results, focusing on their impact on the infectivity of SARS-CoV-2. Finally, in Sec. Conclusions we summarize the benefits of our approach while highlighting its potential limitations and identifying possible future work directions.

## Related work

Since the outbreak of SARS-CoV-2, efforts have been made in order to understand whether certain age groups are more susceptible than others. This could either mean that people from such age groups are more likely to get infected or that they show more severe symptoms compared to older or younger individuals. In addition to this, patients from specific age groups could be more infectious than others, hence more likely vehiculating the disease. To shed light on these problems, viral load—which is a proxy for infectivity—can be a useful tool. It is measured by reverse transcription PCR (RT-qPCR) assays from nasopharyngeal and oropharyngeal swabs via the so-called cycle threshold (Ct) value. The viral load is the virus concentration in the upper respiratory tract and it is usually expressed as the number of viral RNA copies per mL of sample or entire swab specimen or simply by the Ct value.

Several works have analyzed whether viral loads differ between children and adults [1, 15–27], and between young children and adolescents [28–30], and have led to conflicting results. At least eight studies from different countries have concluded that SARS-CoV-2 viral RNA loads among children and adults were comparable [1, 15, 16, 19, 21–24]. In these studies, dependence between age and viral load has been tested using one and two-way analysis of variance (ANOVA) [15] and median of the viral load. A further study [17] found that mean and median viral load values did not vary conspicuously by age but noted that the highest values were measured in patients born from 1995 to 2009. In contrast, at least five studies reported significant differences in viral loads of young children and adults [18–20, 25–27]. Euser et al. [18] suggested an approximately 16-fold higher viral load in the oldest age group (>79 years), compared to the youngest age group (<12 years). Here, age-group differences in the viral load distribution are assessed making use of the Kruskal-Wallis test and linear regression. Another work [20] estimated the amount of SARS-CoV-2 in the upper respiratory tract of young children (<5 years) to be 10-fold to 100-fold greater than in adults whereas a work from Zachariah et al. [30] showed that mean viral load was significantly higher in infants (<1 year) as compared to older children and adolescents.

Finally, one of the largest and most widely followed studies on the subject—even though the number of children and adolescents included is fairly small—was carried out by Jones et al. [9] in early 2020. Dependence between viral load and age has been tested for different age groups both as categorical data and treating age as a continuous variable. In order to compare the viral load of different age categories, the categorical data has been analyzed in a parametric (Welch’s T-test), non-parametric (Mann-Whitney rank test), and Bayesian fashion (modeling viral loads as a mixture of gamma distributions). When considering age as a continuous variable, viral loads have been predicted from age, type of PCR system, and age-PCR system interaction. This study from Jones et al. [9] did not reveal large differences in the viral loads of different age groups, a result that was publicly debated in Germany for its possible implications for school opening policies. In the following, we reanalyze this data. In a very recent publication, Jones et al. [31] extended their initial version of the study. In this newer version, they make use of thin-plate spline regression to conclude that children and adolescents have a slightly lower viral load than adults, but that this difference is unlikely to be clinically relevant.

None of the studies in the literature utilizes the causal framework. In this work, we choose to adopt this framework since it naturally allows to answer the central question of whether the age of a patient causes its viral load and infectivity. It also leads to additional advantages that we will present in the following sections. The problem of deducing causal directions (in particular for the bivariate *x* → *y*, *y* → *x* and *x* ⊥ *y* case) from observational data coming from a joint distribution has been introduced by the works of J. Pearl [32] and P. Spirtes et al. [33], further developed by Mooij, J. et al. [34] and is now a central and non-trivial problem in data analysis. For additional details and motivations behind causal inference theory and techniques we refer to their works.

## Model design

In Sec. Related work we described the state of the art of statistical analyses performed in order to investigate the age dependence of the viral load. These analyses mostly rely on variance tests or correlation assessments. In the following, we motivate the need for a causal model of the viral load distribution. In fact, in order to explain the age and viral load data collected by Jones et al. [9], a well-behaved model should incorporate basic knowledge about the causal relation between age and viral load. It should furthermore allow questioning whether the viral load distribution depends on the patients’ age and quantify the strength of such dependence, if it exists. We consequently develop a non-parametric causal model and apply it to data.

### Motivation

In order to make statements about the factors that contribute to the spread of a disease, we need to rely on data to ground these claims. More importantly, we need to find a model that is capable of identifying and explaining the relationships that underlie the data. Indeed, this should be a minimal requirement for any data-analysis task. The choice of an incorrect model can lead to wrong or misleading conclusions. This is clearly an issue regardless the nature of the data at hand. However, in the case of data describing the pandemic spread the choice of an incorrect model can lead to ineffective or even potentially harmful decisions. It is therefore of vital importance to be able to capture—at least up to a certain level of uncertainty—the interdependences underlying the data.

As discussed in the previous section Sec. Related work, the problem of identifying the relation between age and viral load has been tackled making use of many different techniques. While categorical data analysis and linear correlation analysis struggle to pick up all possible dependences, non-parametric approaches have the drawback of possibly being too (or too little) complicated to account for the actual functional dependence that is inherent to the data. For this reason we want to build a model that is flexible in the sense that it can simply reconstruct independent densities with few degrees of freedom while also being capable of inferring more complex distributions, when needed to describe the data. Moreover, we would like the model to automatically be able to adjust its complexity. This way, the choice of the model is independent from the data analyst’s choices and the results are more consistent and reproducible.

This is another benefit of adopting the causal framework. The concepts of “simple” independence and “more complicated” dependence between variables are natural to this framework and are described by the causal graph and the structural causal model of choice [34]. Furthermore, claiming causal dependence is much stronger than simple linear correlation, which also can arise from a confounder or by mere coincidence. Causal dependence is directional. This is a particularly interesting feature as it allows to test for bias in the data-collection process when a unnatural causal direction is detected from the data analysis, as we will discuss in Sec. Results.

### Causal structure

**Fig 1:**
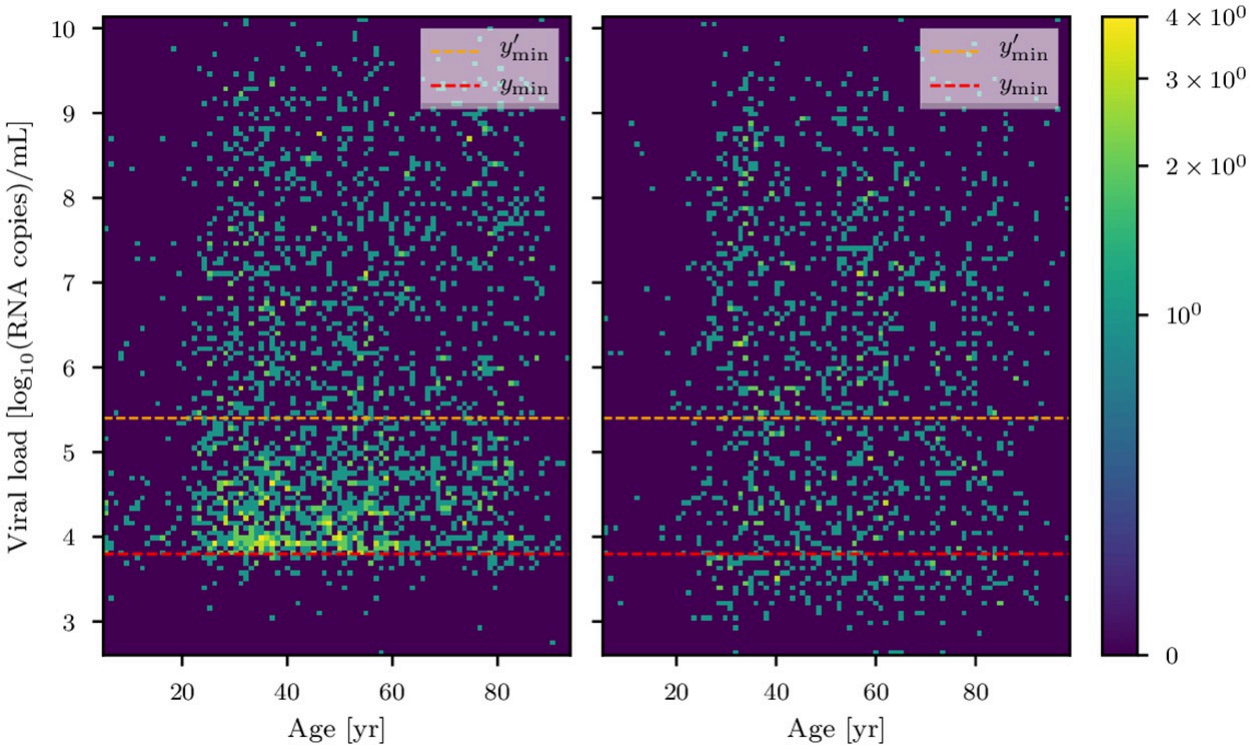
The cobas (left) and the LC 480 (right) datasets. The lower thresholds *y*_min_ and $y_{\text{min}}^{\prime }$ with which the data has been filtered are shown in red and orange, respectively. The number of data counts is color coded with a logarithmic color scheme (see colorbar).

**Fig 2:**
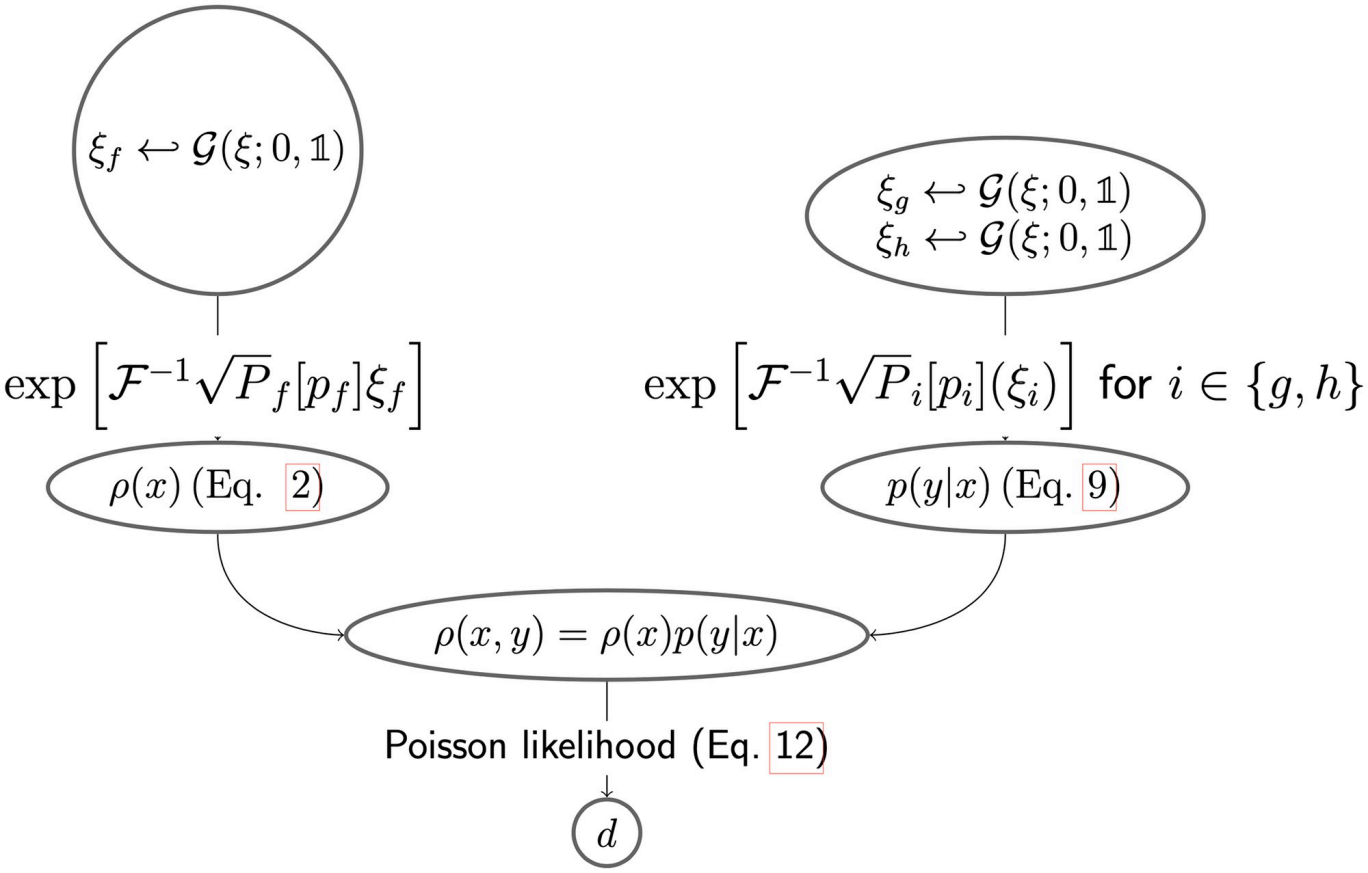
Graph structure of the causal model of age x and viral load y. F denotes the Fourier transform operator. Starting from the top, standard-normally distributed excitations *ξ* are drawn from the latent priors for *f*, *g*, and *h*. They are then transformed into the non-parametric signal *ρ*(*x*, *y*) (Eq 9) by taking the inverse Fourier transform of the Matérn kernel parametrization described in Eq 5. Finally, the signal *ρ*(*x*, *y*) is compared to the data *d* through a Poissonian likelihood (Eq 12).

**Fig 3:**
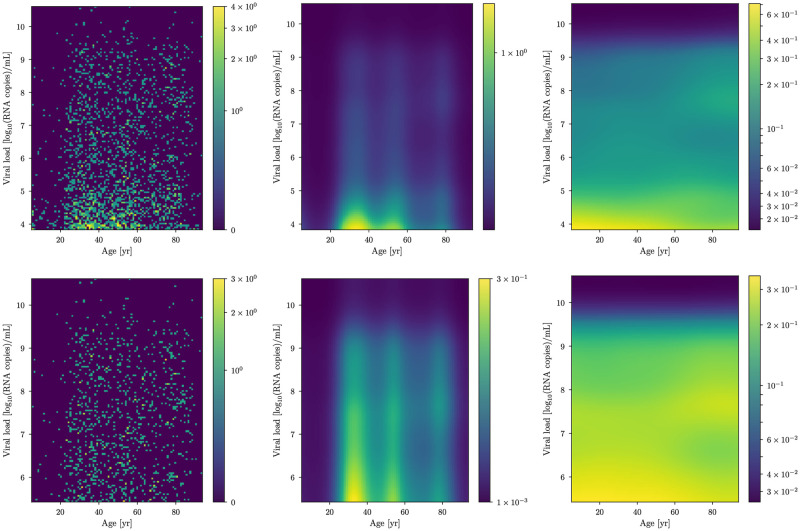
Cobas dataset analysis. Left: The cobas dataset. Middle: the reconstructed density distribution *ϱ*(*x*, *y*) as a function of the age (*x*) and the viral load (*y*) in a logarithmic coloring scheme. Right: The 2D conditional probability distribution *p*(*y*|*x*) of the viral load (Eq 9) obtained by fitting the model. The data and the results of the analysis are shown for two different data-filtering thresholds *y*_min_ (top) and $y_{\text{min}}^{\prime }$ (bottom).

**Fig 4:**
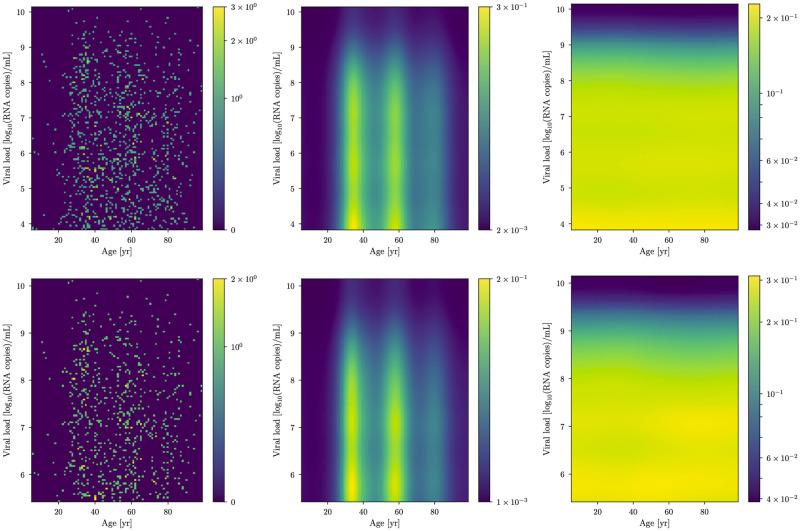
LC 480 dataset analysis. The same as in Fig 3, but for the LC 480 dataset.

The analyzed dataset $d={\left\{\left(i,x_{i},y_{i}\right)\right\}}_{i=1}^{N}$ consists of indexed pairs of age *x*_*i*_ = age_*i*_/years and log-viral load *y*_*i*_ = log_10_(viral RNA copies_*i*_/ml) values for each of the *N* infected patients, extracted from Fig. 6 in [9]. The dataset is shown in Fig 1. For simplicity, we refer to *y* as the “viral load”, where 10^y^ are the number of viral RNA copies per milliliter of sample or entire swab specimen. We assume that the data points are Poisson process counts drawn from an underlying stationary density distribution *ϱ*(*x*, *y*). Using the causal model shown in Fig 2 we reconstruct the density *ϱ*(*x*, *y*). Our final density reconstructions are displayed in Figs 3 and 4. To model this density, we express it in terms of the underlying age distribution *ϱ*(*x*) of the patients times the conditional PDF *p*(*y*|*x*) of the viral load *y* given the patient’s age *x*,

$$\begin{array}{c} \varrho (x,y)=\varrho (x)\;p(y|x). \end{array}$$ (1)

In Eq 1 the causal direction *x* → *y* (age influences viral load) is implicitly introduced. Even though *x* → *y* might appear to be the most intuitive causal direction, since the immune system reaction depends on age and thus age should affect the viral load, we note that selection effects could introduce different apparent causal structures in the data. If the data had been collected in such a way that the viral load (*y*) was the deciding factor for whether a patient would enter the data sample, with an age (*x*) dependent threshold, the viral load would impact the age distribution in the sample, leading to an apparent *y* → *x* causal structure.

As a practical example, one could imagine physicians to request a PCR test for a child only if their symptoms were worse compared to those of an adult for which they would have requested a PCR test. Since this and similar selection effects cannot be fully excluded for the analyzed data (see discussion in [9]), we will calculate the Bayesian evidence for the possible causal relations *x* → *y*, *y* → *x*, and *x* ⊥ *y* (i.e. *x* and *y* are independent, *p*(*y*|*x*) = *p*(*y*)). As a final remark, we note that other effects such as, e.g., delays in data reporting, could in principle also be confounding the simple *x* ⇆ *y* causal direction. Li and White [35] have for instance shown how reporting delays can have an effect on the pandemic-spread models. Effectively, a confounding variable *z* would influence both *x* and *y*, namely *x* ← *z* → *y*. Although we cannot completely exclude the presence of such a confounding variable, the randomization tests that we describe in Subsec. Outline support our implicit assumption of absence of any leading confounding variable.

To model the *x* → *y* causal direction, we need to model the age distribution *ϱ*(*x*) of the infected patients according to the causal structure introduced in Eq 1. Lacking knowledge on the exact details of the patient selection process, we assume *ϱ*(*x*) to be a log-normally distributed random variable. The log-normal distribution is a natural choice since an age density is by definition a strictly positive and continuous quantity. Another natural assumption is the absence of abrupt changes, since no sharp age-selecting processes are expected to have shaped it. We fulfill these assumptions with the choice

$$\begin{array}{c} \varrho \left(x\right)=\varrho_{0}e^{f(x)}, \end{array}$$ (2)

where *ϱ* 0 = *N*/100 is a reference density and f:[0,100]↦R a smooth function centered around zero. We accordingly assume *f* to be drawn from a zero centered Gaussian process with a prior covariance *F*

$$\begin{array}{c} P\left(f\right)=G\left(f,F\right)≔\frac{1}{\sqrt{2\pi F}}\text{exp}(-\frac{1}{2}f^{†}F^{-1}f). \end{array}$$ (3)

The covariance

$$\begin{array}{c} F_{xx^{\prime }}={\left\langle f\left(x\right)\;f\left(x^{\prime }\right)\right\rangle }_{\left(f\right)}≔\int Df\;P\left(f\right)\;f\left(x\right)\;f\left(x^{\prime }\right) \end{array}$$ (4)

determines the degree of smoothness of the logarithmic distribution function, as well as the characteristic length scale and the amplitude of its variations. We assume this correlation structure to be translation invariant *F*_*xx*′_ = *F*(*x* − *x*′), since we only expect it to depend on age differences—and not on a particular age value—and parametrize it with a Matérn kernel. Invoking the Wiener-Khinchin theorem, we can represent such a translation-invariant correlation function in Fourier space with a spectral density of

Pfk=af21+k/kf2γf2, (5)

with *a*_*f*_ specifying the amplitude of the variations in *f*, 1/*k*_*f*_ the characteristic length-scale above which the variations become uncorrelated, and *γ*_*f*_ the spectral index, which determines the smoothness of the variations. We infer all three covariance parameters *p*_*f*_ ≔ (*a*_*f*_, *k*_*f*_, *γ*_*f*_) from the data. In order to ensure that the model is flexible enough to fit the data, we set mildly informative priors on the covariance parameters. We denote by $P(f|p_{f})$ the probability of a specific realization of *f* given the Matérn kernel parameters *p*_*f*_, as described by Eqs 3 to 5.

Next, we have to specify the distribution of the viral load given the age, *p*(*y*|*x*). To do so, we note that we can directly model an independent distribution for which *p*(*y*|*x*) = *p*(*y*) in the same way as we modeled *ρ*(*x*), i.e. by choosing *p*(*y*|*x*) ∝ *e*^*g*(*y*)^. Of course, *p*(*y*|*x*) can in general exhibit an arbitrary complicated dependence on *x*. We model any additional complicated entanglement between the age *x* and the viral load *y* with a new function *h*(*x*, *y*). By doing so, we introduce a degeneracy between *g*(*y*) and *h*(*x*, *y*), since they can both model *y*-only dependent structures. In principle, the function *h*(*x*, *y*) can in fact model any function *p*(*y*|*x*) without the necessity of introducing *g*(*y*). To solve this problem, we choose

$$\begin{array}{c} p\left(y|x\right)\propto \frac{e^{g(y)+h(x,y)}}{\int e^{h(\tilde{x},y)}d\tilde{x}}. \end{array}$$ (6)

To ensure that only *g*(*y*) models strictly *y*-dependent features and that all possibly complicated *x*−dependent features are captured by *h*(*x*, *y*), we have to prevent *h*(*x*, *y*) from modeling any strictly *y*-dependent structure that has been already captured by *g*(*y*). We do this with the denominator $\int e^{h(\tilde{x},y)}d\tilde{x}$ in Eq 6, that removes from *p*(*y*|*x*) any *y*-only structure contained in *h*(*x*, *y*), hence eliminating the degeneracy between *g*(*y*) and *h*(*x*, *y*).

We can verify that *h*(*x*, *y*) indeed satisfies the desired property of not encoding any structure that could be represented by *g*(*y*) by simply substituting *h*(*x*, *y*) ↦ *h*′(*x*, *y*)≔ *h*(*x*, *y*) + *g*′(*y*) in Eq 6, where *g*′(*y*) depends only on *y*. This results in *h* and *h*′ leading to the same conditional PDF

p′(y|x)∝egy+h′x,y∫eh′x˜,ydx˜=egy+hx,y+g′yeg′y∫ehx˜,ydx˜=egy+hx,y∫ehx˜,ydx˜∝p(y|x) (7)

and we can therefore conclude that only *g*(*y*) can model strictly *y*-dependent features.

As desired, for vanishing *h* it still holds

$$\begin{array}{ccc} {p(y|x)}_{h(x,y)=0} & \propto & e^{g(y)}\propto p\left(y\right), \end{array}$$ (8)

which implies independence, *y* ⊥ *x*. Thus, a non-trivial *h*(*x*, *y*) models the *x* → *y* causal influence while a trivial *h*(*x*, *y*)≡0 represents causal independence. We now want to make sure that the more complicated *x*−dependence modeled by *h*(*x*, *y*) is only introduced if it is strictly needed to explain the data. This way, we can clearly distinguish the causal scenarios *x* → *y* or *y* → *x* from the independent *x* ⊥ *y* scenario. In fact, the distinction between *g* and *h* would be meaningless without a prior choice that favors independence between *x* and *y*. Hence, we assume *g* and *h* to be drawn from zero-centered Gaussian processes. In this way, without any information coming from the data, the most likely realizations of both of these functions are the identically-zero functions *g*(*y*) = *h*(*x*, *y*) ≡ 0. However, if the data exhibits strictly *y*-dependent features, meaning that the marginal distribution *p*(*y*) is non-trivial, these features can only be represented by a non-zero *g*(*y*).

Strictly *y*-dependent features are indeed clearly visible in the data. For example, we notice that in Fig 3 higher viral loads are by far more rare than lower viral loads. In this case, the data-inferred *g*(*y*) is non-zero and shows this decreasing feature. Following the same reasoning, the most likely distribution for *h*(*x*, *y*) in absence of data is identically-zero everywhere. Again, *h*(*x*, *y*) will only be non-zero in case that the data triggers some coupling between *x* and *y*. Thus, the model favors independence of *x* and *y* (by favoring *h* ≡ 0) and the inferred density will be entangled in *x* and *y* only in the case the data exhibits this feature. This shows how the level of complexity of the model is adapted to the data automatically, without having to make any additional explicit model choice.

We again assume a Matérn-kernel-shaped correlation structure for the Gaussian process *g*, with covariance parameters *p*_*g*_ ≔ (*a*_*g*_, *k*_*g*_, *γ*_*g*_). We set the priors on *p*_*g*_ as for *p*_*f*_ and learn these parameters from the data as well. Since the typical length scales and amplitudes of the variations of *h*(*x*, *y*) in *x* and *y* directions are not in principle a priori similar, we assume the covariance for *h* to be shaped by a direct product of individual Matérn kernels in the *x* and *y* directions. For their corresponding prior parameters, $p_{h}=\left(p_{h}^{\left(x\right)},p_{h}^{\left(y\right)}\right)$ with $p_{h}^{\left(i\right)}=\left(a_{h}^{\left(i\right)},k_{h}^{\left(i\right)},\gamma_{h}^{\left(i\right)}\right)$ and *i* ∈ {*x*, *y*}, we use similar hyper-priors as before, i.e. as for *p*_*f*_ and *p*_*g*_, respectively. For more details on the prior choices we refer to the S1 Appendix. We call the ensemble of all these kernel parameters *p* ≔ (*p*_*f*_, *p*_*g*_, *p*_*h*_). The details on how the Gaussian process for *h* with a Matérn kernel product covariance structure is set up is described in the S1 Appendix, where we describe a multi-dimensional density estimator that is agnostic to causal directions.

Lastly, we normalize the conditional PDF

$$\begin{array}{c} p\left(y|x\right)=\frac{e^{g(y)+h(x,y)}}{\int e^{h(\tilde{x},y)}d\tilde{x}}\;{\left(\int \frac{e^{g\left(\tilde{y}\right)+h\left(x,\tilde{y}\right)}}{\int e^{h(\tilde{x},\tilde{y})}d\tilde{x}}d\tilde{y}\right)}^{-1} \end{array}$$ (9)

such that the full model density reads

$$\begin{array}{c} \varrho \left(x,y\right)=\varrho_{0}e^{f(x)}\;\frac{e^{g(y)+h(x,y)}}{\int e^{h(\tilde{x},y)}d\tilde{x}}\;{\left(\int \frac{e^{g\left(\tilde{y}\right)+h\left(x,\tilde{y}\right)}}{\int e^{h(\tilde{x},\tilde{y})}d\tilde{x}}d\tilde{y}\right)}^{-1}. \end{array}$$ (10)

A schematic representation of the forward causal model is shown in Fig 2. The assumed causal structure *x* → *y* is introduced in the model by the asymmetry between the roles of the *x* and *y* coordinates and the zero-centered Gaussian process priors on *f*, *g*, and *h*. Interchanging *x* and *y* leads to a model that follows the opposite causal direction *y* → *x*. This allows to empirically distinguish these causal directions by calculating the model evidences for the two opposite scenarios, namely *x* → *y* and *y* → *x*, as well as to test for *x* ⊥ *y* by enforcing *h* = 0.

The evidence already inherenty penalizes more complicated models (see [36]), i.e. models that require a higher number of degrees of freedom. Using the ELBO as a criterion for model selection has therefore the additional benefit of regularizing the solutions. In other words, the evidence further helps the data analyst to univocally pick the lowest complexity model which can best explain the data, in addition to the already self-adjusting level of complexity provided within the model itself.

Finally, we note that the choice of *x* and *y* is up to now completely arbitrary. Therefore, the method described in this work can be used to assess the causal direction of any continuous two-dimensional data distributions, given the data and some loose (prior) information about the correlation structure. the proposed method is thus very general and can be used for different datasets and analysis schemes with respect to those described in this paper. For example, it could be used to test the causal relation between the concentration of greenhouse gasses in the atmosphere and the average global temperature rise in Celsius degrees.

### Likelihood

In order to construct the likelihood P(d|ϱ(·,·)), we bin the data into a fine two dimensional grid over the *x* and *y* coordinates with 90 × 128 pixels, such that

$$\begin{array}{c} n_{ij}\left(d\right)=\sum_{m=1}^{N}\int_{i\Delta x}^{(i+1)\Delta x}dx\int_{j\Delta y}^{(j+1)\Delta y}\delta \left(x-x_{m}\right)\delta \left(y-y_{m}\right)dy \end{array}$$

contains the number of cases within the (*i*, *j*)^th^ pixel of size Δ*x* = 1 yr and Δ*y* ≃ 0.04 log_10_(viral RNA copies_*i*_)/ml. These counts *n*_*ij*_ are then compared with the model’s expectations

$$\begin{array}{c} \begin{array}{cc} \lambda_{ij} & ≔\lambda_{ij}\left(\varrho \right)=\int_{i\Delta x}^{(i+1)\Delta x}dx\int_{j\Delta y}^{(j+1)\Delta y}\varrho \left(x,y\right)dy\\ & \approx \Delta x\Delta y\;\varrho (()(()i+\frac{1}{2})\Delta x,(()j+\frac{1}{2})\Delta y) \end{array} \end{array}$$ (11)

via a Poisson likelihood

$$\begin{array}{c} P\left(d|\varrho \right)=\prod_{i,j}\frac{\lambda_{ij}^{n_{ij}}}{n_{ij}!}e^{-\lambda_{ij}}. \end{array}$$ (12)

### Inference

The full model involving the data *d* as well as all the unknown quantities, which compose the signal vector *s* ≔ (*f*, *g*, *h*, *p*), reads

$$\begin{array}{c} \begin{array}{cc} P(d,s) & =P(d|s)\;P(s),\;\text{where}\\ P(d|s) & =P(d|\varrho [f,g,h])\;\text{and}\\ P(s) & =P\left(f|p_{f}\right)P\left(g|p_{g}\right)P\left(h|p_{h}\right)\;P\left(p_{f}\right)\;P\left(p_{g}\right)\;P\left(p_{h}\right). \end{array} \end{array}$$ (13)

At this stage, we need to convert our causal model into an inference machine for the signal vector *s*. We do this conversion by reformulating the model in the language of information field theory [10, 11], transforming the coordinates of the signal vector *s* = *s*(*ξ*) such that the prior on the new *ξ* coordinates becomes an uncorrelated Gaussian P(ξ)=G(ξ,1) as described by Knollmüller and Enßlin [37]. We then implement the resulting model using the Python package Numerical Information Field Theory (NIFTy) [38–40] and finally use NIFTy’s implementation of Metric Gaussian Variational Inference (MGVI) [41] to approximate the posterior distribution in the new coordinates

$$\begin{array}{c} P\left(\xi |d\right)=\frac{P(d|\xi )P(\xi )}{P(d)}\approx G\left(\xi -{\bar{\xi }}_{d},\Xi_{d}\right) \end{array}$$

with a Gaussian which has posterior mean ${\bar{\xi }}_{d}$ and covariance Ξ_*d*_, where the *d* suffix indicates the dataset used in the inference. This Gaussian posterior encodes the approximate result of the inference in the new coordinates. In order to translate this into the signal coordinates, we have to transform P(ξ|d) to P(s|d) using the relation *s* = *s*(*ξ*). This relation is non-linear and the resulting PDF is neither Gaussian nor practical to obtain analytically. In order to evaluate moments from the posterior distributions of the desired quantities, MGVI provides *ξ*-samples drawn from the approximate Gaussian posterior $\xi ↩G(\xi -{\bar{\xi }}_{d},\Xi_{d})$. These *ξ*-samples can be converted via the coordinate transformation *s* = *s*(*ξ*) into the signal space, where they represent (approximate) signal posterior samples. Making use of these posterior samples it is possible to calculate the posterior expectation values and model uncertainties of any desired quantity *q*(*s*):

$$\begin{array}{c} \begin{array}{cc} \bar{q} & ≔{\left\langle q\left(s\right)\right\rangle }_{\left(s|d\right)}\approx \frac{1}{N_{\text{s}}}\sum_{i=1}^{N_{\text{s}}}q\left(s\left(\xi_{i}\right)\right)\\ \sigma_{q}^{2} & ={\left\langle {\left(q\left(s\right)-\bar{q}\right)}^{2}\right\rangle }_{\left(s|d\right)}. \end{array} \end{array}$$ (14)

Here, *ξ*_*i*_ denotes the *i*^th^ of the *N*_s_ drawn samples. In particular, the posterior mean of the conditional PDF *p*(*y*|*x*) and of any quantity which can be calculated from *p*(*y*|*x*) can thereby be obtained, as well as the resulting uncertainties characterized via their uncertainty dispersion. In general, these posterior signal samples will not follow Gaussian statistics because the transformation is typically non-linear. Furthermore, since MGVI is a variational inference approach, the calculated uncertainties will be slightly smaller compared to the ones given from the accurate posterior. However given the complexity and size of the model, we need to use an approximate inference method as MGVI. For details about this methodology, as well as extensive performance and accuracy tests, we refer to Knollmüller and Enßlin [41].

### Identifying causal directions with the Evidence Lower Bound

We now focus on understanding the causal relations given by the interplay between the variables. The proposed causal model should allow to discriminate between all possible causal structures, namely *x* → *y*, *y* → *x*, and *x* ⊥ *y*. Since we have a different model for each of these causal directions, we can use the Bayesian evidence to select the model that is better suited to the data. We do this once again by exploiting our variational inference scheme. It has been shown that the evidence is indeed a consistent and robust criterion for model selection [42, 43].

First, we need to define the independent model, i.e. a model for which age and viral load are regarded as statistically independent variables. Such a model is built by setting a very tight zero-centered prior on *h*, hence removing it from the model for all practical purposes. We can then estimate the Bayesian evidence both for the causal–hence dependent—models (*x* → *y* and *y* → *x*) and compare it with the evidence of the independent model (*x* ⊥ *y*). More precisely, we calculate the so called Evidence Lower Bound (ELBO) as a proxy for an evidence. Using the posterior uncertainty covariance Ξ as well as the posterior samples provided by MGVI, we can compute the ELBO [36] for each model. This is lower than the exact logarithm of the evidence by the information difference (as measured in nits) between the exact and approximated posterior of a model. If too much information is not lost during MGVI, the ELBO should be a good approximation of the exact log evidence. Furthermore, we can assume that deviations from the exact log evidence should be similar among different models, thereby reducing the effect of the MGVI approximations on differences between log-evidences. Thus, we can use the ELBO as a good proxy for the log model evidence ratios of similar models. The stochastic sampling steps performed in order to estimate the ELBO introduce a sampling uncertainty. This uncertainty can be in principle reduced by taking more samples, at the expense of larger computational costs. We state this numerical one-sigma uncertainty for all MGVI and ELBO based log-evidences. The *y* → *x* model is obtained by swapping the coordinates of the *x* → *y* model. The quantity of interest is then the logarithm of the evidence ratio of each causal model with the independent one,

$$\begin{array}{c} \Delta E_{x⇆y}=\text{log}\frac{p(d|x⇆y)}{p(d|x⊥y)}, \end{array}$$ (15)

where *x* ⇆ *y* denotes either *x* → *y* or *y* → *x*. Δ*E*_*x*→*y*_ indicates the log evidence in favor of the causal model *x* → *y* with respect to the independent one and similarly Δ*E*_*y*→*x*_ the one for *y* → *x*. Comparing Δ*E*_*x*→*y*_ with Δ*E*_*y*→*x*_ also allows to discriminate between the two possible causal directions in the dataset. We identify the preferred causal direction underlying the data in subsec. Causal directions and bias, where we also discuss its implications.

## Data

We make use of RT-PCR viral load data collected from the Charité Institute of Virology and Labor in Berlin, Fig. 6 in Jones et al. [9]. The data was acquired with two different PCR instruments, Roche cobas 6800/8800 (cobas dataset, which we denote by *d*_C_ and is comprised of ≈2200 data points) and Roche LightCycler 480 II (LC 480 dataset, which we denote by *d*_L_ comprised of ≈1350 data points). In the following, we will show the difference between the two datasets. As can be seen from the count difference in the raw data plots as of Fig. 6 of [9] and in the histogramed data in Figs 3 and 4, for low viral loads (*y* ≲ 5 in units of log_10_(RNA copies/ml)), the LC 480 dataset shows a roughly uniform count distribution in the whole viral load domain. In contrast, the cobas dataset exhibits an increasing number of counts in the *y* ∈ [2.0, 3.8] viral load domain followed by a descending trend in counts in the *y* ∈ [3.8, 5.0] region. For this reason and in order to better understand the possible shortcomings of both instruments, we analyzed the data in two different ways.

Since the major differences between the datasets arise for viral load values *y* ∈ [3.8, 5.0] we define two lower thresholds for the viral load (*y*_min_ ≔ 3.8 and $y_{\text{min}}^{\prime }≔5.4≃{\text{log}}_{10}\left(250000\right)$ in units of log_10_(viral RNA copies/ml)) and discard any data point for which the viral load is lower than *y*_min_ and $y_{\text{min}}^{\prime }$, respectively. We set the lower threshold *y*_min_ at the value for which the number of counts of the cobas dataset is maximum (see Fig 1). Below this threshold, the counts’ density lowers dramatically. As for $y_{\text{min}}^{\prime }$, the value of 250000 in units of viral RNA copies/ml indicates the threshold for the isolation of infectious virus in cell cultures at more than 5% probability as described by Wörfel et al. [44]. We then analyze the cobas and LC 480 datasets first neglecting the data below *y*_min_ threshold and then below $y_{\text{min}}^{\prime }$. For the sake of simplicity, we will denote the cobas and LC 480 datasets with *y*_min_ and $y_{\text{min}}^{\prime }$ as a lower threshold as *d*_C_, $d_{\text{C}}^{\prime }$, *d*_L_, and $d_{\text{L}}^{\prime }$, respectively. This way we can highlight the differences between the two datasets and investigate possible sources of systematic errors in the viral load measurement instruments or different selection effects in the data collection process.

The datasets are not explicitly provided by the authors. Therefore, we acquired the data by means of a plot digitizer algorithm from Fig. 6 of [9]. Since the age coordinates are not labeled precisely, but only a rough interval Δ_age_ ∼ 0 − 100 yr is provided, we do not expect the acquired data points to be accurate—especially in the age domain. For this reason the obtained age axis could be affected by a global shift up to 5 − 10 years in any direction. But, since our model is translation invariant, this kind of systematic bias in the data extraction process (an overall shift in the age values for all data points) does not affect the causal inference machinery. Nonetheless, the reader should keep this in mind when interpreting the results concerning the conditional PDF of the viral load given the age. Concerning the viral load coordinates, for which more precise units were given, the data should be regarded as more reliable. For our aim of building a method to analyze age and viral load data in a non-parametric and causal fashion providing uncertainty estimates, this level of accuracy is sufficient. The results we present from here on are given for the measured coordinate values without considering any uncertainty with respect to the real quantities (age, viral load). Nevertheless, we do not believe systematic or random error contributions in the data extraction procedure to significantly affect our results since the shapes of the learned distributions are translation invariant.

## Results

In the following, we discuss the main results and their relevance for the infectivity of different age groups.

### Age dependence of the viral load

For both lower thresholds and datasets *d*_C_, *d*_L_, we estimate the model parameters *s* by means of the MGVI algorithm implemented in NIFTy. In Figs 3 and 4 we show the data (left panel) and the correspondent reconstructed underlying densities *ϱ*(*x*, *y*) (central panel).

**Fig 5:**
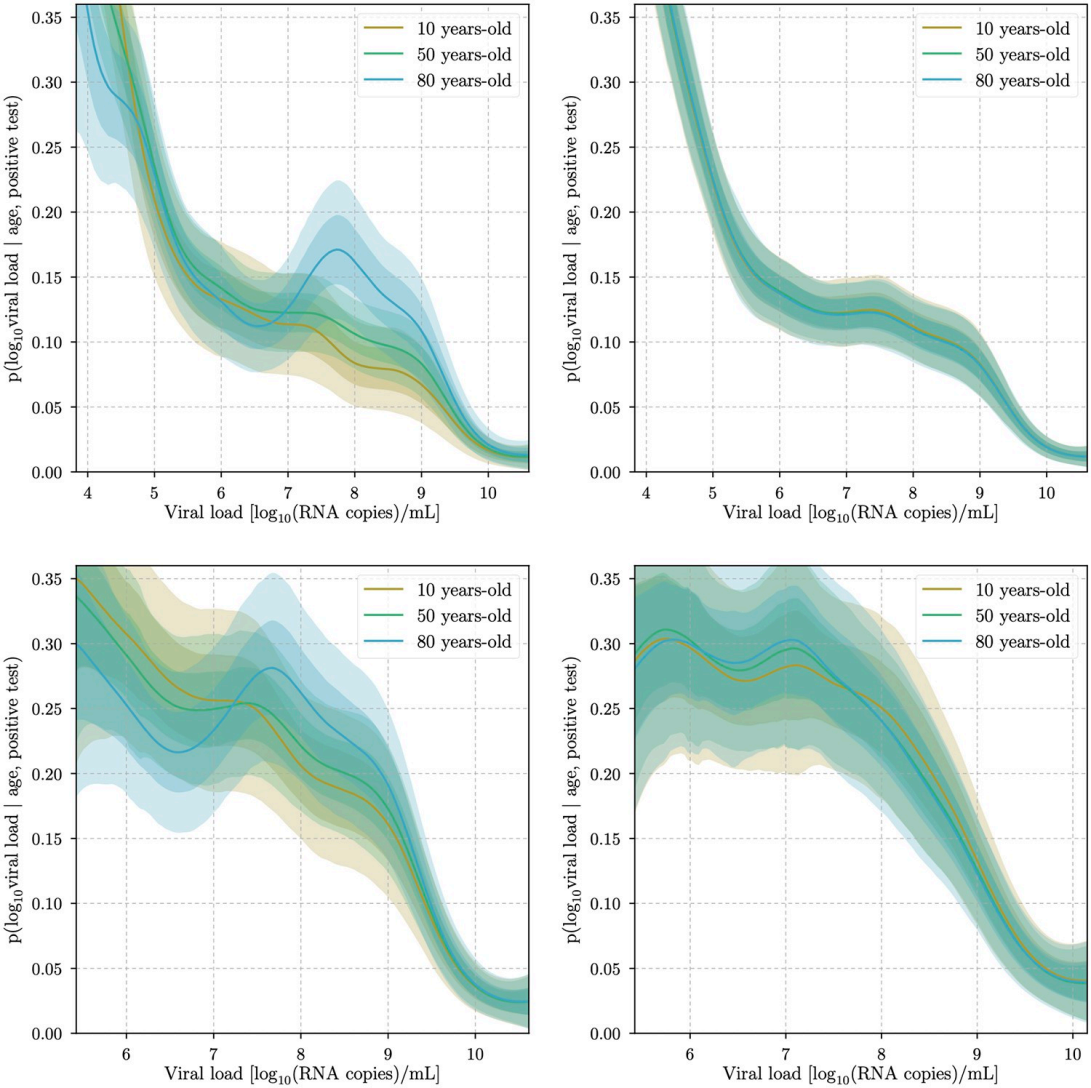
Viral-load probabilities for specific ages. Viral load conditional PDF *p*(*y*|*x*) for specific ages *x* ∈ {10, 50, 80}. Panel one and two (top) display the results for the cobas dataset *d*_C_ and for the randomized cobas dataset *d*_C_ respectively. The latter serves as a null test, since the randomization erases any (causal) relation between the age (*x*) and the viral load (*y*) other than shot noise. Panel three and four (bottom) show *p*(*y*|*x*) for the cobas dataset $d_{\text{C}}^{\prime }$ and for the LC 480 dataset $d_{\text{L}}^{\prime }$ respectively, both with the higher viral load threshold $y_{\text{min}}^{\prime }$. The shaded regions represent 1*σ* and 2*σ* uncertainty contours of the approximate posterior.

A multi-modal age distribution is clearly visible as well as an overall decrease for growing viral loads in all of the reconstructed densities. The conditional PDF *p*(*y*|*x*) of the viral load *y* given the age *x* is shown in Fig 5. In the conditional probability, the multi-modal age structure displayed by the density is not visible since it has been absorbed by *ϱ*(*x*). What this effectively means is that age-only selection effects—e.g., testing one or many specific age groups more than others or demographics in general—have been modeled, and the resulting conditional probability distribution does not depend on such effects.

For all datasets and ages, a general descending trend in the viral load probability distribution is clearly visible. The reconstruction based on the *d*_C_ dataset exhibits significant differences in the viral load for different ages (Fig 5, first panel). For infected patients approximately above the age of 60, the distribution exhibits a distinct maximum for viral loads of *y* ≃ 8 (in units of log_10_(RNA copies/ml)). Furthermore, we show that this feature is indeed triggered by the data, and is not just the result of an over-fit of sample noise. To do so, we apply a random permutation *r* to the viral load values in the *d*_C_ dataset, the only one that exhibits a possible *x* → *y* causal structure. We then analyze the randomized dataset $d_{\text{C}}^{r}={\left\{\left(i,x_{i},y_{r(i)}\right)\right\}}_{i=1}^{N}$ in the same way as seen for $d_{\text{C}}={\left\{\left(i,x_{i},y_{i}\right)\right\}}_{i=1}^{N}$. The resulting conditional PDF *p*^*r*^(*y*|*x*) reconstructed from the randomized dataset (Fig 5) does not exhibit any clear age-dependent structure in the viral load, indicating that the differences seen in the real data are not just a shot noise effect.

### Causal directions and bias

As discussed in Subsec. Causal structure, we can use ELBO ratios to identify causal relations. In our analysis, this corresponds to choosing between the *x* → *y*, *y* → *x*, and *x* ⊥ *y* models. Identifying the causal direction underlying the data will allow to show that selection effects distorting the expected causal relation *x* → *y* are subdominant and that for the cobas dataset *d*_C_ we indeed see evidence for an age dependence of the viral load distribution, *p*(*y*|*x*) ≠ *p*(*y*).

As a matter of fact, the log-evidence ratio for the *d*_C_ dataset is Δ*E*_C,*x*→*y*_ = 4.6 ± 1.0 for *d*_C_, which clearly favors the dependent model, but this value decreases to $\Delta E_{\text{C},x\to y}^{\prime }=-1.5\pm 1.0$ when considering $d_{\text{C}}^{\prime }$, hence $y_{\text{min}}^{\prime }$ as a lower threshold. We highlight that a log-evidence difference between the two compared models of 1 unit corresponds to a factor of *e* ≈ 2.7 for the Bayesian odds ratio between the two. Thus, Δ*E*_C,*x*→*y*_ = 4.6 ± 1.0 implies given equal model priors, *p*(*x* → *y*) = *p*(*x* ⊥ *y*), a posterior model odds ratio of $p\left(x\to y|d\right):p\left(x⊥y|d\right)=e^{3.5\pm 0.7}\approx 99.5_{\left[36.6\right]}^{\left[270.4\right]}$ in favor of a causal dependence between viral load and age for the cobas dataset with the lower threshold *y*_min_.

For the opposite causal direction we get Δ*E*_C,*y*→*x*_ = −0.2 ± 1.1, which shows that there is no strong *y* → *x* structure in the data. For the LC dataset *d*_L_ these evidence differences with respect to the independent model become Δ*E*_L,*x*→*y*_ = −4.6 ± 1.0 and $\Delta E_{\text{L},x\to y}^{\prime }=-3.4\pm 1.0$ respectively for the two thresholds. Therefore the independent model is favored in both cases. This shows that the cobas ($d_{\text{C}}^{\prime }$) and LC 480 ($d_{\text{L}}^{\prime }$) datasets are in agreement for viral loads which are higher than $y_{\text{min}}^{\prime }$, but in case we include the data lying in the viral load region $y\in [y_{\text{min}},y_{\text{min}}^{\prime }]$, the causal age-dependent structure becomes visible in *d*_C_ and is not negligible anymore.

It is known that model evidences can vary strongly with different data realizations. Moreover, in order to calculate the evidences, we invoked approximations and stochastic calculation steps. Thus, proper null-tests are required in order to validate and calibrate the evidence ratio calculation. We provide such null-tests by repeating the data randomization step described above several times, thereby producing many randomized datasets. By construction, these dataset should not exhibit any causal structure. Indeed, for the 10 randomized-dataset realizations performed, we find much lower log-evidence ratios between dependent (causal) and independent models with respect to the ones found for the original cobas dataset *d*_C_. The average difference between these tests is 〈Δ*E*_random,*x*→*y*_〉_randomizations_ = −6.0 ± 1.0. Since none of the randomized dataset realizations reaches comparably high log-evidence ratios with respect to the independent model, all these findings support robustly the argument that the dependent model is a more suited description of the *d*_C_ dataset. As previously discussed, this result also supports the assumption that no additional variable *z* is confounding *x* ← *z* → *y* the bivariate causal direction *x* ⇄ *y*. Had there been a strong confounder *z*, we would have expected a more complicated data distribution that could have possibly been detected when comparing the actual data with the randomized datasets. Even if this did not appear to be the case, we cannot completely rule out the presence of a weak confounder.

**Fig 6:**
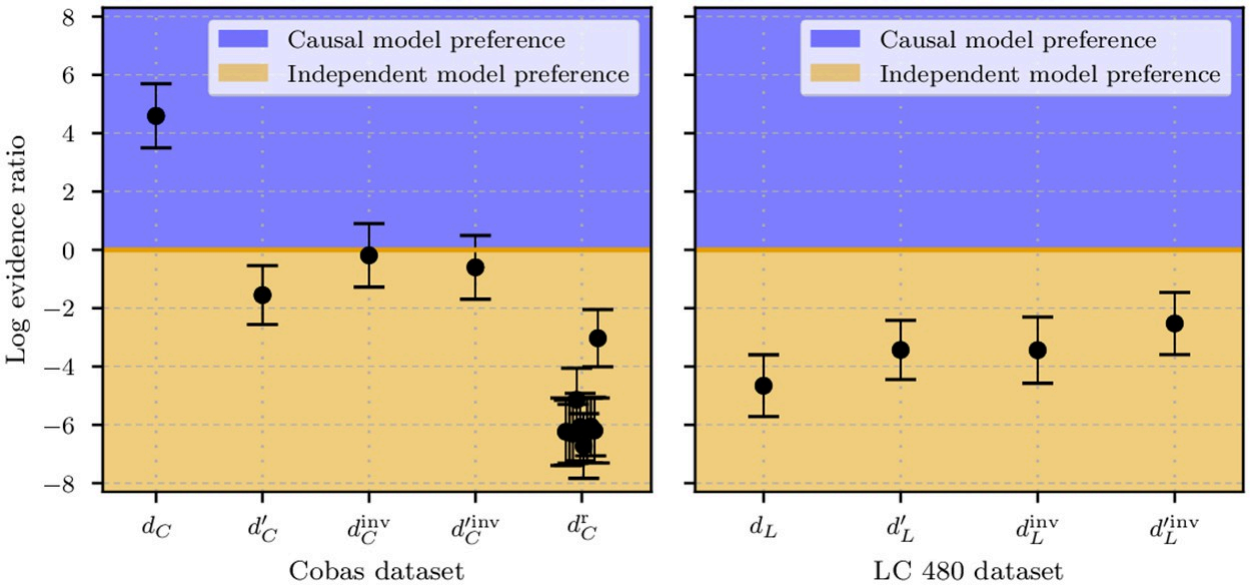
Evidence results. Natural logarithm of the evidence ratio with respect to the correspondent independent model for the *x* → *y* cobas datasets *d*_C_ and $d_{\text{C}}^{\prime }$, for the *y* → *x* causal model $d_{\text{C}}^{\text{inv}}$ and ${d^{\prime }}_{\text{C}}^{\text{inv}}$ (left panel). The log-evidence ratios labeled with $d_{C}^{r}$ display the (*y*-)randomized datasets (for 10 different realizations). The same is also shown for the LC 480 dataset (right). The error bars represent the numerical uncertainty associated to the stochastic estimate of the ELBO. A positive logarithm of the evidence ratio denotes a preference for the given causal model with respect to the correspondent independent model and vice versa. The strength of this preference can be determined by taking the exponential of the log-evidence ratio between the causal model and the correspondent independent one. For example, a log-evidence ratio of 5 corresponds to a *e*^5^ ≈ 141-fold preference for the examined causal model.

The results of the evidence calculations are displayed in Fig 6. This figure also indicates that the independent model interpretation of the data is favored for all other datasets and threshold combinations (except for *d*_C_), since the evidence for the independent model is always higher.

These contradicting results for the two datasets (or thresholds) *d*_C_ and $d_{\text{C}}^{\prime }$ might have several possible explanations. First, they could be caused by a potential accuracy loss of the PCR devices below certain viral load values, as suggested for the cobas dataset *d*_C_ below $y_{\text{min}}^{\prime }$ in [9], hence for the Roche cobas 6600/8800 PCR system. It could also mean that the opposite is happening and the Roche LC 480 PCR device is less sensitive than the Roche cobas 6600/8800 in the *y* ∈ [3.8, 5.0] region. This possibility would be supported by the fact that the cobas dataset exhibits a clear age dependent structure in such viral load region, but the (swab) data processed by the cobas PCR device contains no information on the patients’ ages. Hence it would be surprising that a systematic effect in the measurements could introduce an age dependence on the viral load distribution. Furthermore, this pattern–that older patients exhibit higher viral loads—is plausible from a medical perspective.

Nevertheless, we cannot exclude the possibility that selection effects have been introduced in the data. We have already shown that “viral load causing age” effects (*y* → *x*) are subdominant. Nevertheless, selection effects could still have been introduced for instance by collecting age and viral load subsamples from a “viral-load biased” population sample. This could happen, e.g., if symptomatic patients had been predominantly tested. Since children are less likely to show symptoms than adults, the sample would then include mainly those children with higher viral loads.

And finally, a combination of such competing effects could have affected the results and thereby imprinted a spurious age dependence to the viral-load distribution. Given only the statistical data in our possession, this possibility cannot be ruled out completely.

### Impact on infectivity

**Fig 7:**
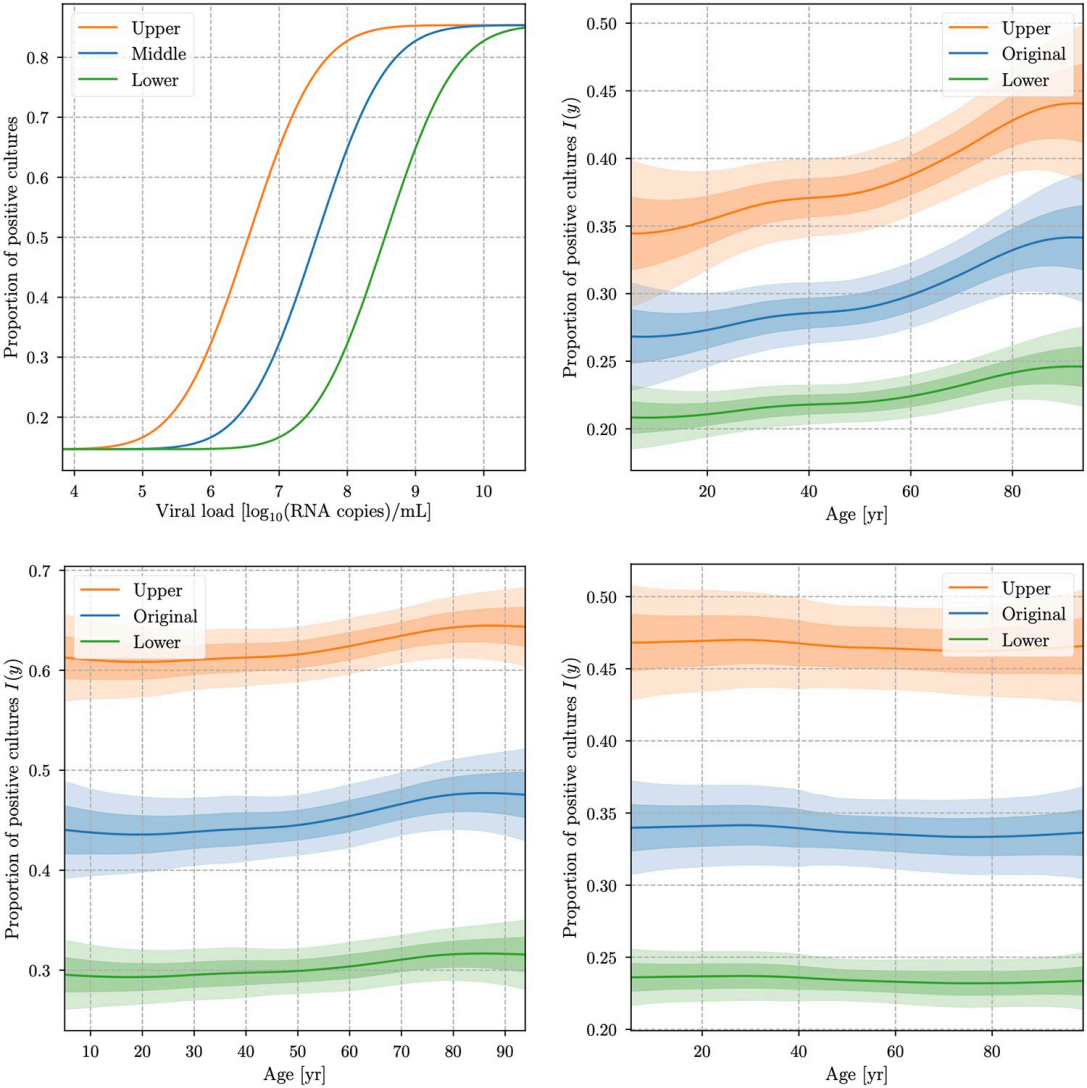
Infectivity proxy vs. age. *I*(*y*) obtained from Fig. 1g in [44] and fit with a probit function (top left, Original), then projected for different patients’ ages for the cobas dataset *d*_C_ (top right), $d_{\text{C}}^{\prime }$ (bottom left) and for the LC 480 dataset *d*_L_ (bottom right). The upper and lower curves are obtained by translating *I*(*y*) of one unit in viral load as in *I*(*y* − 1) and *I*(*y* + 1) in order to give upper and lower bounds similar as those shown in Fig. 1g in [44]. The shaded regions show 1*σ* and 2*σ* uncertainty contours of the approximate posterior.

After having established a potential age difference in the viral load distribution for *d*_C_, we investigate whether this difference—if real—would be relevant for the infection dynamics. For this purpose, we need to link the viral load to the infectivity *I*(*y*) of the virus, i.e. the probability of transmitting the infection. Infectivity can be measured in different ways. In our analysis, we choose the projected virus isolation success based on probit distribution described in [44] as a proxy for infectivity. This represents the infection success rate for cell cultures exposed to saliva with different viral load *y* and can be seen as the blue curve in Fig 7, labeled with “Original”. We will from now on refer to this parameter as “infectivity proxy” and indicate it with *I*(*y*).

The projected infectivity as a function of the age is then given by the expectation value of the infectivity parameter over the conditional PDF *p*(*y*|*x*)

$$\begin{array}{c} I\left(x\right)≔{\left\langle I\left(y\right)\right\rangle }_{p(y|x)}=\int I\left(\tilde{y}\right)p\left(\tilde{y}|x\right)d\tilde{y}. \end{array}$$ (16)

The result, together with the uncertainties resulting from our PDF modeling are shown in Fig 7. No relative differences larger than **0.18** for the (projected) infectivity of the different age groups is found, with typical values of *I*(*x*) ≈ 0.3 for all datasets. This means that at most a 50% difference in infectivity due to different viral load between different age groups—but more likely a smaller one—should be expected.

In order to characterize the uncertainty resulting from our *I*(*y*) modeling, due to the uncertainty of the original determination of this function and due to the uncertainty in the identification of viral loads with different instruments, we repeat the analysis while shifting the original *I*(*y*) curve by one order of magnitude upwards and downwards in *y*. The resulting maximal relative difference in the infectivity of the different age groups is ≃ 0.3. Thus, even though our model allows us to show that infectivity exhibits an age dependence if the ***d*_C_** dataset with *y*_min_ provides a valid picture, the viral load differences between different age groups though are not strong enough to impact on the infection dynamics at a level that justifies regarding any age group as noninfectious or even significantly less infectious.

## Conclusions

In order to investigate the controversial results reported in the literature, we developed a causal model to assess the dependence of viral loads of patients infected with COVID-19 on age. The developed model is capable of reconstructing two-dimensional density distributions from data counts and to learn causal directions. The model complexity is set in a user-independent fashion making the results more robust and consistent. Furthermore, its causal nature allows to make predictions about non-directly measured quantities (e.g. the infectivity assessment in Sec. Results and Fig 7) and to additionally test for bias in the data-collection process.

Although the benefits of a causal analysis were already discussed in Pearl’s original work [32] and are usually well recognized, causal inference is not so commonly applied to real-world data science, often because it requires the implementation of complicated ad-hoc models. With the hope of making it useful for a wide range of data-science applications we make our causal model freely available. This model is very flexible and generic (as it only requires a set of *x* and *y* data pairs and mild priors on their correlation structures) and it can therefore be used in future epidemiological studies as well as in completely different fields. We provide the source code under an open source license for usage in further studies and applications at https://gitlab.mpcdf.mpg.de/ift/public/causal_age_viral_load_model. As a side product, we also developed a causal-direction-agnostic density estimator, which is described in more detail in the S1 Appendix.

Using our novel method to model causal relations non-parametrically, we have reanalyzed the SARS-CoV-2 age and viral load data presented in [9]. In doing so, we have found statistically significant differences in the viral load distribution of different age groups when regarding the cobas dataset *d*_C_ for viral loads within the interval of 10^3.8^ to 10^5.0^ in units of viral RNA copies/ml of sample or entire swab specimen as reliable. These differences become irrelevant if this region is ignored in the analysis.

We cannot completely exclude that selection effects in the data-collection process may have introduced an apparent causal relation between viral load and age, but the observed trend—a statistically-significant increase in the viral load with age—fits with the generally accepted notion that the immune system response gets weaker with age. Assuming this trend to be real, we showed, however, that its expected impact on the infectivity of different age groups is at most moderate. For this reason we cannot exclude any age group from being considered as a potentially significant source of infection.

The region of the cobas dataset relevant for this trend is described in [9] as containing an artifact, suggesting that the correct interpretation of the data is that viral load, hence infectivity, is predominantly age independent. Here, we want to point out that other studies on the age dependence of the viral load present in the literature [15, 18] make the opposite claim. Moreover, in their most recent publication, Jones et al. [31] acknowledge an age dependence of the viral load. This dependence is quantitatively similar to the one we have detected with our method. Furthermore, the causal evidence tests presented in Sec. Results favor considering the age dependence of the viral load as a real effect and not just as an artifact. These tests also disfavor the reverse-causal-direction model (here: that the viral load of a patient “causes” its age), which would indicate that strong selection effects have affected the data-collection process. We introduced these tests as a new tool to detect potential systematic effects in similar datasets.

In conclusion, the results of our analysis ultimately confirm some of the findings in the literature—i.e. that the viral load is only modestly dependent on the age—but with a much higher sensitivity and robustness. Of central importance are the methods here developed. While being tailored to describe the Covid-19 pandemic data, they can be easily adapted for more general purposes and can prove very useful also for future pandemics or for new—and possibly more infective—mutations of SARS-CoV-2.

## Supporting information

S1 Appendix Matérn-kernel density reconstruction. (PDF)
