# Supplementary material for: Causal, Bayesian, & non-parametric modeling of the SARS-CoV-2 viral load distribution vs. patient’s age
Source: PLoS One. 2022 Oct 5;17(10):e0275011. doi: 10.1371/journal.pone.0275011 (PMC9534394; doi:10.1371/journal.pone.0275011)
Supplement: S1 Appendix — (PDF) [file pone.0275011.s001.pdf]

Supporting Information to: Causal, Bayesian, & Non-parametric Modeling of the SARS-CoV-2 Viral Load Distribution vs. Patient's Age

Matteo Guardiani[1,2,*], Philipp Frank[1,2], Andrija Kostić[1,2], Gordian Edenhofer[1,2], Jakob Roth[1,2], Berit Uhlmann[4], and Torsten Enßlin[1,2,3]

1 Max Planck Institute for Astrophysics, Garching, Germany
2 Fakultät für Physik, Ludwig-Maximilians-Universität München, Munich, Germany
3 Excellence Cluster ORIGINS, Garching, Germany
4 Süddeutsche Zeitung, Munich, Germany

* matteani@mpa-garching.mpg.de

Supporting information

S1 Appendix. Matérn-kernel density reconstruction. We aim to infer an unknown distribution from a random realization of discrete data. In the following, we present a general-purpose density estimator that serves this scope. Using a fully Bayesian framework, we can propagate uncertainties through each inference step and extract information from the correlation structure inherent to the data. We provide our method as open-source software.

According to Chen, Tareen, and Kinney [1], the problem of extracting a smooth density function from a limited set of data samples is a challenging and well-known problem in statistical learning and data analysis. The most common ad-hoc methods to empirically derive (probability) densities from data usually involve histograms or Kernel Density Estimation (KDE) [2, 3]. These methods do not infer the smoothness of the learned (probability) density's correlation structure and are thus prone to reconstructing unphysical densities. Other methods make use of neural networks (see for example Liu et al. [4]) or restrict the density to specific functional forms (see e.g. Dirichlet Process Mixture Model, or DPMM [5–7]). Another approach is to use smooth priors and infer the level of smoothness of the reconstructed density from data via maximum entropy (Density Estimation using Field Theory, or DEFT [8, 9]). Chen, Tareen, and Kinney [1] propose an interesting information-theoretical-based modification of DEFT, although it only effectively works in one dimension. Finally, another very commonly used and effective solution to the density estimation problem is the one given by deep neural networks. In a recent work, Liu et al. [4] propose a generative adversarial networks (GAN) which is particularly effective in high dimensions. We refer to their work for comparison with similar neural-network-based approaches.

Most of these approaches lack a robust estimate for uncertainties or specify none at all. In the hope of addressing these shortcomings, we propose our novel general-purpose density reconstruction method, which we will refer to as Matérn Kernel Density Estimator (MKDE). This method is very general, works for a generic n-dimensional space, and therefore applies to many different contexts and fields. We presented one paradigmatic example of its many possible applications in Subsec. Causal Structure. In this example, we used MKDE to reconstruct the continuous distributions of the ages and viral loads of patients infected with Covid-19 from age-and-viral-load data samples collected within the general population.

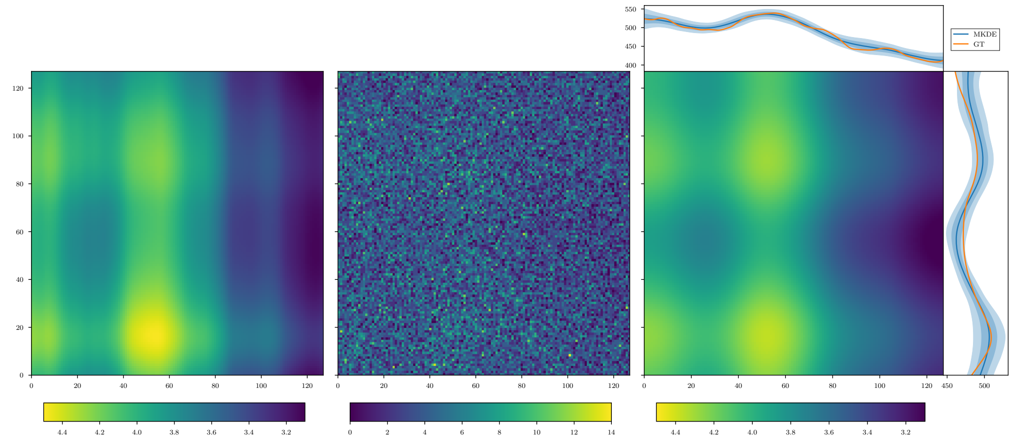

Fig 1. Example of MKDE performance. Left: random realization of a smooth probability density with independent covariance structure in the x and y direction (ground truth, GT). Middle: Poissonian counts drawn from the GT. Right: MKDE reconstruction from the counts. In the same panel, we also show the marginals of the reconstructed density (blue lines), together with their one and two-sigma uncertainty estimates (shaded areas in blue) against the marginals of the GT (orange lines).

MKDE is capable of reconstructing a smooth density distribution underlying an – even limited – discrete dataset. We achieve this result under the hypotheses that the data points are drawn from the underlying density through a Poisson process and that the reconstructed density is a sufficiently smooth function. Since we expect the inferred (probability) density to be strictly positive and to vary on logarithmic scales, we choose the log-normal model

$$\varrho(x) = e^{s(x)}, \tag{1}$$

where $s(x)$ is the natural logarithm of the density. In the case of multi-dimensional data, both the density $\varrho(x)$ and its logarithm $s(x)$ in Eq. 1 are functions defined over n-dimensional vector spaces, with n being the dimension of the data space (e.g. space, time, age, viral load, ...). For the sake of simplicity, we will initially show how a one-dimensional density is reconstructed, emphasizing how to generalize to the multi-dimensional case only when this generalization is non-trivial.

Smoothness is a common and ubiquitous assumption when dealing with physical data. To fulfill this assumption in the MKDE, we parametrize the two-point correlation structure of the Gaussian process that determines the value of the log-density $s(x)$ at each point and for each dimension with a Matérn kernel. This kernel is a very flexible choice [10]. Moreover, it is very well suited to represent a priori homogeneous covariance structures like the ones we want to model. We define the Gaussian process $s := s(x) = (s_x)_{x\in\mathbb{R}} \hookleftarrow \mathcal{P}(s) = \mathcal{G}(s, S)$ on the one-dimensional position space $\mathbb{R}$. This process has a homogeneous covariance structure $S_{xx'} = C_s(x - x')$ that can be efficiently represented in Fourier space thanks to the Wiener-Khinchin theorem. Invoking this theorem, we can use the power spectrum $P_s(k)$ to fully determine the Fourier transform of the two-point correlation function $C_s(x - x')$ for any stationary and statistically homogeneous process, and in particular for the Gaussian process s.

In order to draw prior samples from the Gaussian field s, we choose a standardized coordinate system $\hat{\xi}$. We then transform the standard normally distributed parameters $\xi = (\xi_k)_k$, where $k \in \mathbb{N}$ is the Fourier-space index according to the mapping

$$s = \mathcal{F}^{-1}\, A\, \xi \quad \text{with } A := \mathrm{diag}(\sqrt{P_s}). \tag{2}$$

Here, $\mathcal{F}$ represents the Fourier transform operator and A the amplitude operator in Fourier space. The amplitude operator encodes the Matérn-kernel correlation structure, parametrized with

$$A_{kk'} = 2\pi\,\delta(k-k')\frac{a_s}{[1+k^2/k_0^2]^{-\gamma_s/4}}, \quad (3)$$

where a_s is a scale factor which accounts for the standard deviation in position space, k_0 is the magnitude of the characteristic correlation-length wavevector, and γ_s is the spectral index of the power spectrum. We assume a_s and k_0 to be a priori log-normally distributed since we expect strictly positive variations of the possible power spectra on a logarithmic scale. Similarly, we choose γ_s to be normally distributed, since the spectral index could in principle also be negative. We additionally introduce volume factors to ensure that the model parameters are intensive with respect to volume, i.e. they do not depend on the volume in position space.

For higher-dimensional data, we expect the correlation structure along each axis (or dimension) to be a priori independent from the others. These different axes could in fact have very different meanings (and units), as they could represent – for instance – space and time, temperature, pressure, and volume, age and viral load (as seen in Subsec. Causal Structure, or a different combination of these and other continuous quantities. Therefore, in the n-dimensional data space we can decompose the amplitudes of the correlation structure

$$A_{k,q,\ldots,n} = \bigotimes_{i\in\{k,q,\ldots,n\}} A_i \quad (4)$$

along each independent axis, each modeled by an individual amplitude operator A_i, for $i \in \{k, q, \ldots, n\}$. The zero modes of the individual axes must be treated separately in order to avoid degeneracy. Thus, in the proposed model the zero mode is shared among all directions and inferred independently through an a priori strictly-positive and uniformly-distributed parameter α. For more details on the zero mode degeneracy and factorizing power spectra, we refer to Arras et al. [11].

At this stage, we can summarize all the parameters that we have introduced for each independent axis with the scalar-valued parameters α, a_s, k_0, and γ_s and the vector-valued ξ_k. We set broad priors on these parameters and learn them using MGVI. We set the following priors on the signal parameters: $\alpha = [10^{-15}, 5.0]$, $a_s = (0.3 \pm 0.2)$, $k_0 = (4.0 \pm 3.0)$, and $\gamma_s = (-6.0 \pm 3.0)$, where the mean and standard deviation specify a Gaussian prior distribution for γ_s and log-normal distributions with the given mean and standard deviation for a_s and k_0. For details on the inference of posterior estimates for the MKDE parameters through MGVI and their uncertainty quantification, we refer to Subsec. Inference Fig. 1 illustrates the performance of MKDE in a two dimensional setting.

In conclusion, we described MKDE, a Matérn-kernel-based, Bayesian, and non-parametric density estimator that can construct a smooth (probability) density function from an - even limited - set of data samples. The broad priors on the learned parameters, combined with the log-normal model and the Matérn kernel covariance structure, make MKDE very flexible and robust. Furthermore, the Bayesian inference framework allows for posterior uncertainty quantification for the reconstructed density. A software implementation of MKDE is available in NIFTy 7 and is also released as an open-source Python package (DENSe), which can be found at: https://ift.pages.mpcdf.de/public/dense/.

Priors. Throughout the analysis, we have made the use of the following priors on the signal parameters: $a_f = (0.3 \pm 0.1)$, $k_f = (5.0 \pm 3.0)$ yr^{-1}, and $\gamma_f = (-3.0 \pm 2.12)$,

where the mean and standard deviation specify a Gaussian prior distribution for γ_f and log-normal distributions with the given mean and standard deviation for a_f and k_f.

References

1. Chen WC, Tareen A, Kinney JB. Density Estimation on Small Data Sets. Phys. Rev. Lett.. 2018;121(16):160605. doi:10.1103/PhysRevLett.121.160605.

2. Silverman BW. Density estimation for statistics and data analysis. vol. 26. CRC press; 1986.

3. Sheather SJ. Density Estimation. Statistical Science. 2004;19(4):588–597.

4. Liu Q, Xu J, Jiang R, Wong WH. Density estimation using deep generative neural networks. Proceedings of the National Academy of Sciences. 2021;118(15).

5. Ferguson TS. A Bayesian Analysis of Some Nonparametric Problems. The Annals of Statistics. 1973;1(2):209 – 230. doi:10.1214/aos/1176342360.

6. Müller P. Bayesian nonparametric data analysis. Cham, Switzerland: Springer; 2015.

7. Gelman A. Bayesian data analysis. Boca Raton: CRC Press; 2014.

8. Kinney JB. Estimation of probability densities using scale-free field theories. Phys Rev E. 2014;90:011301. doi:10.1103/PhysRevE.90.011301.

9. Kinney JB. Unification of field theory and maximum entropy methods for learning probability densities. Phys Rev E. 2015;92:032107. doi:10.1103/PhysRevE.92.032107.

10. Genton MG. Classes of Kernels for Machine Learning: A Statistics Perspective. J Mach Learn Res. 2002;2:299–312.

11. Arras P, Frank P, Haim P, Knollmüller J, Leike R, Reinecke M, et al.. M87* in space, time, and frequency; 2021.
